# Fostering resilience in young people with intellectual disabilities using a ‘settings’ approach

**DOI:** 10.1177/17446295231168186

**Published:** 2023-03-28

**Authors:** Sandy Whitelaw, Anthony Bell, Ailsa Mackay, Heather Hall

**Affiliations:** School of Interdisciplinary Studies, 3526 University of Glasgow, Dumfries, UK; School of Interdisciplinary Studies, 3526 University of Glasgow, Dumfries, UK; National Centre for Resilience, School of Interdisciplinary Studies, 3526 University of Glasgow, Dumfries, UK; The Usual Place/Inspired Community Enterprise Trust Ltd (ICET), Dumfries, UK Inspired Community Enterprise Trust Ltd (ICET)

**Keywords:** resilience, health promotion, settings-based approaches

## Abstract

The need to foster resilience amongst young people with intellectual disabilities is increasingly recognised within policy. Critically, understanding of the actual means by which this aspiration might be most sensitively and effectively met is considered weak. This paper reports on an exploratory case-study of a social enterprise community café – *The Usual Place* - that through the promotion of employability, seeks to promote resilience amongst its young ‘trainees’ with intellectual disabilities. Two research questions were set: “*how is ‘resilience’ conceptualized within the organisation*” and “*what features within the organisation are significant in fostering resilience”?* We identify a range of significant features associated with being able to successfully foster resilience – the need for a foundational ‘whole organisation’(settings) approach based on high levels of participation and choice; the negotiation of a constructive dynamic tension between ‘support’ and ‘exposure’; and the embedding of these actions in embodied actions and day-to-day organisational activities.

## Background

Our paper reports on an exploration of the nature of organisational dynamics in a local social enterprise community café based in rural south-west Scotland, *The Usual Place* (TUP) that were associated with efforts to foster resilience. The organisation works with young people with intellectual disabilities (‘trainees’), specifically seeking to enhance their employability through a nuanced mix of café work placements, intensive needs-led support and the attaining of externally accredited vocational qualification (‘*Scottish Vocational Qualifications*’). This work in turn is associated with the broader desire to promote health and wellbeing amongst these individuals.

Beyond simply facilitating access to existing healthcare services (Alborz et al., 2005), this aspiration of actively *promoting* health and wellbeing in general and resilience specifically amongst those with intellectual disabilities is increasingly recognised within policy (Scottish Government, 2013), practice (Taggart and Cousins, 2014) and research (Boland, Daly, and Staines, 2008). It has been articulated in relation to the need for ‘health promotion’ that addresses both physical issues associated with ‘healthy *lifestyles*’ such as diet, smoking, physical activity and substance misuse (Roll, 2018) and more recently, the emergence of a focus on *mental* health as a domain of interest (Naaldenberg et al, 2013). Of particular significance to this paper, the growing significance of the associated concept of ‘resilience’ is also noteworthy within this mentail health domain (Scheffers, van Vugt and Moonen, 2020).

Critically, some feel that this these fields and subsequent interventions have tended to be framed in a relatively narrow and rudimentary fashion - around *individualistic* behaviour change (Scott and Havercamp, 2016) and as a form of *self-efficacious* resilience expressed as individual ‘coping’ and ‘adaptation’ (Mackay, Shochet and Orr, 2017). This has been considered problematic in two ways: first, that generic programmes tend not to be adapted to the particular needs of those with intellectual disabilities (Truesdale and Brown, 2017); second, in of themselves, such approaches are relatively ineffective in the context of this group (Naaldenberg et al, 2013). Consequently, this has led some to point out that, “the evidence supporting these theories in people with disabilities is sparse” (Cardell, 2015; 1).

Driven by the WHO’s general (the Ottawa Charter, 1986) and mental health specific (Friedli, 2009) influences, a ‘reframing’ of this ground has however emerged (Cardell, 2015) in three main respects. First, the significance of ‘context’ for health has been stressed (Kuijken et al, 2020). Second the concept of resilience has been highlighted (Scheffers, Moonen and van Vugt, 2022).Third, the impact of deeper social determinants on health (Frier et al, 2018) and again resilience (Emerson, 2013) for those with intellectual disabilities is recognised. In practical terms, these perspectives have informed the emergence of various progressive models – particularly, ‘*public health*’ (Association of Young People’s Health, 2016), ‘*whole systems*’ (Fredman, 2006) and ‘*settings*’ (Vlot-van Anrooij et al, 2020b) based approaches to promoting health (Vlot-van Anrooij, 2020c) and resilience (Scheffers, van Vugt and Moonen, 2020) for people with intellectual disabilities. We will develop these ideas in more detail later, but for now and in brief, all suggest that the attainment of *individual* outcomes is strongly determined by wider *social* and *organisational* contextual determinants and that these features can be critical in affecting any manifestation of resilience.

Whilst offering novel and progressive insights, some feel that these circumstances have tended to be couched in relatively vague and aspirational terms (Whitelaw et al, 2001), particularly lacking detail of how the *theoretical* ideals contained within the domains might be *practically* expressed and implemented (Dooris, Wills and Newton, 2014). Insights from previous work that explored the fundamental nature of TUP (Whitelaw et al, 2021) had suggested that the ‘real world’ nature of this social enterprise setting *might* implicitly be associated with this more grounded and experiential approach to promoting health and resilience in the potentially demanding yet regulated and supportive circumstances that exist in any working business. So, in this context, TUP was considered a productive case study to examine this practical potential and consequently, further work was commissioned by the Scottish Government’s *National Centre for Resilience* to explore this possibility.

This pilot project [for a full report on the work see NCR (2022)] sought to answer the following research questions: *how is ‘resilience’ broadly conceptualized within the setting (TUP)?*; and *what features of the TUP as a setting are significant in fostering resilience and what barriers exist?* We start the paper by briefly setting out some key conceptual resources that underpinned our empirical work. We then outline the methodological base of the work before describing our field insights related to both of our research questions and associated theoretical context. We conclude by reflecting more broadly on the potential for the pragmatic use of a settings-based approach to foster resilience amongst young people with intellectual disabilities.

## Key concepts

Based on a scoping literature review, our empirical work (and subsequent analysis and reporting) was informed by a series of inter-related theoretical resources associated with ‘resilience’ in general (e.g. Theron and Theron, 2014), a ‘settings’ based approach to resilience (e.g. Vlot-van Anrooij et al, 2020b), specifically in the context of young people with intellectual disabilities (e.g. Panicker and Chelliah, 2016).

In terms of essential conceptualisations of resilience, Michael Ungar’s ecologically framed approach to resilience soon became apparent and significant (Ungar, 2008). This approach focusses on the inter-relationship between individuals and their physical and social environments - the significance of *both* potentially antagonistic *deterministic contexts* (WHO, 1986) but also the possibility of *individual agency* as personal adaptability and ‘bouncing back’ from difficult life experiences (WHO, 2021). Ungar’s contention that resilience is, *“the capacity of individuals to navigate their way to health-sustaining resources, including opportunities to experience feelings of well-being, and a condition of the individual’s family, community and culture to provide these health resources and experiences in culturally meaningful ways*” (Ungar, 2008; 225) therefore became a central theoretical touchstone for our work.

More specifically, Ungar et al’s (2007) notions of ‘navigation’, ‘negotiation’, and ‘tensions’ were also highly relevant: *navigation* referring to how young people find their way to resources that foster resilience; *negotiation* highlighting the importance of these resources being appropriate and contextually accessible; and of most significance to our work, various *tensions* that define the interconnection between culture, context and individual agency in manifesting resilience. Ungar et al. (2007; 305) proposes that these complex systemic interactions lead to resilience being the consequence of a series of complex ‘*unique pathways*’. These resources are set out below.

Developing further the basis of Cardell’s (2015) ‘reframing’ introduced above and initial thoughts on ecological approaches, a series of additional, more detailed perspectives are significant. As a form of transition into thinking about the pragmatic basis of potential interventions, a potential variance is recognised between seeing resilience as the product of minimising and fixing ‘deficits’ and alternatively, one that focusses on the possibility of promoting ‘strengths’, *independent* of deficiencies (Christmas and Khanlou, 2019). On balance, most tend to suggest a preference for adopting the latter rather than the former approach - in relation to both individual attributes and ‘protective’ contextual features (Zimmerman, 2013; Fergus and Zimmerman, 2005) and this is justified both theoretically/psychologically/ethically in that it is preferable to promote assets than ‘fix’ deficits (Chu, Saucier and Hafner, 2010) and pragmatically, in that evidence suggests that such work tends to be more effective (Leve et al, 2012). This ‘asset’ orientation is also associated with two further concepts in the literature: the centrality of a positive ‘enablement’ orientation (Ungar and Theron, 2020) in fostering resilience; and the notion that an ‘embodied’ approach to the functional manifestation of resilience (Rajan-Rankin, 2014).

Extending this ‘solution’ based analysis, two further related features were noticeable in the literature; the general notion of a ‘whole community’ approach to resilience (Plodinec, Edwards and Whitde, 2014); and the specific concept of a ‘healthy settings’ approach (Dooris, 2013). For example, Khanlou and Wray (2014) proposes the notion of an ecologically informed ‘whole community’ approach to fostering resilience that has the potential to access “a wide range of powerful social influences and relationships that foster and promote the development of resilience […] that cut across individual, family, community, school and society” (Khanlou and Wray, 2014; 75).

These foundational ecological and settings based principles have recently found particular expression in the emergence of ‘settings’ approaches specifically related to those with intellectual disabilities (Vlot-van Anrooij et al, 2020a; Vlot-van Anrooij et al, 2020c). This ground notes the particular significance of physical and social *environments* for those with intellectual disabilities (Vlot-van Anrooij et al, 2020c) and suggests the potential for the fostering of resilience to be associated with various cultural and procedural organisational features that are both “co-created” and then “embedded in the day-to-day practices of ID care organizations” (Vlot-van Anrooij et al, 2020c; 2).

## Methodology

Drawing on guidance from similar studies within the resilience field (e.g. Beltman et al, 2020; Henderson et al, 2021; McGee, 2013; Jefferis and Theron, 2017; Fourie and Theron, 2012; Kumpulainen et al, 2016; Mabhoyi and Seroto, 2019; Bezuidenhout et al, 2018), we decided to adopt a phenomenological approach to this study. This research tradition emphasises the importance of “how individuals process experience in their everyday lives” (Hesse-Biber, 2017; 26), making it a particularly suitable theoretical approach for this project in that it allowed us to understand how individuals and groups processed and conceptualised ‘resilience’ within a specific social setting.

**Figure 1.**
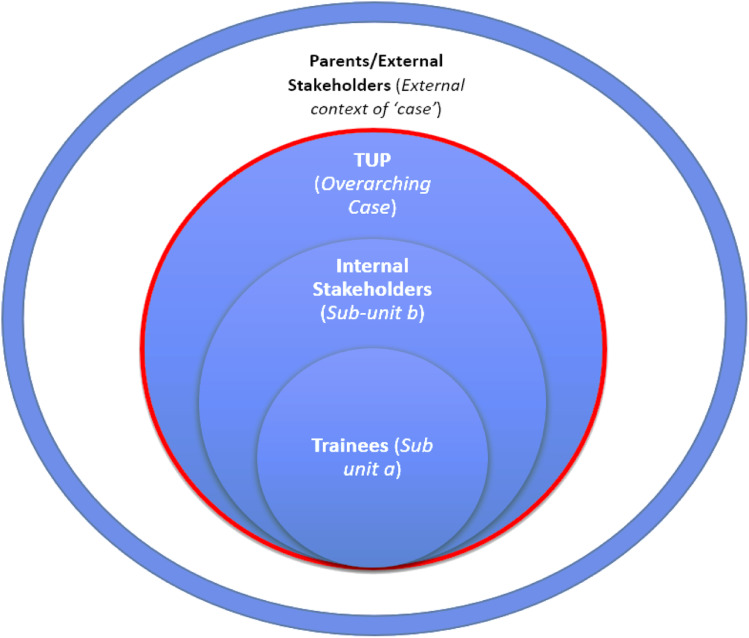
The Bounded Concept of The Usual Place Case.

Furthermore, an ‘exploratory’ (Yin, 2017; 15) and ‘organisational-based’ (Vincent and Wapshott, 2014) case study approach was deployed. The over-arching unit of the case was TUP as a whole organisation. Furthermore, based on the orientation set out above that saw the fostering of resilience as a complex process involving an interplay between varying contextual and interpersonal factors (Theron, Murphy and Ungar, 2021), an “embedded unit design” (Baxter and Jack, 2008; 550) was used where a series of identified initial overarching organisational sub-elements (such as, organisational structure, leadership, organisational cultures and grounded work practices) articulated with a range of groupings: TUP staff (both leadership and on-the-ground practitioners); the young people themselves (‘trainees’); and external stakeholders (parents of the trainees and a representative from a national level disability advocacy group). This conceptualisation is outlined in Figure 1 below.

Within this context, a purposive sampling approach was adopted (Bowling, 2014) complemented by an element of opportunism within these groups - in relation to being able to access TUP staff and trainees within the busy nature of the day-to-day work in the café. The sample is summarised in Tables 1 and 2 below

**Table 1.** Ungar’s seven tensions amended from Ungar et al (2007).

Tension	Example
1. Access to material resources	Availability of financial, educational, medical and employment assistance and/or opportunities, as well as access to food, clothing and shelter
2. Relationships	Relationships with significant others, peers and adults within one’s family and community
3. Identity	Personal and collective sense of purpose, self-appraisal of strengths and weaknesses, aspirations, beliefs and values, spiritual and religious identification
4. Power and control	Experiences of caring for one’s self and others; the ability to effect change in one’s social and physical environment in order to access health resources
5. Cultural adherence	Adherence to one’s local and/or global cultural practices, values and beliefs
6. Social justice	Experiences related to finding a meaningful role in community and social equality
7. Cohesion	Balancing one’s personal interests with a sense of responsibility to the greater good; feeling a part of something larger than one’s self socially and spiritually

**Table 2.** The project sample.

Sub-unit	N. of participants	Occupation/role breakdown
External stakeholders (parents, external professionals connected to TUP)	5 (2 took part in the focus group)	2 parents, 3 external associated professionals
Internal stakeholders (staff members)	5 (2 took part in the focus group)	3 senior staff members, 2 mentors
Trainees	7	All trainees at TUP

Fieldwork was conducted within TUP between December 2021 and March 2022, using three methods. First, based on similar resilience-based work conducted by Mabhoyi and Seroto, (2019), Atkinson (1998) and Theron and Theron (2014), *narrative life-course interviews* with TUP trainees were deployed (see Supplemental appendix 1). Second, founded on similar resilience-based work conducted by Kumpulainen et al, (2016), Fourie and Theron (2012) and Thompson et al (2020), *semi structured stakeholder interviews* were used (see Supplemental appendix 2). Finally, drawing on similar resilience-based work conducted by Angevaare et al, 2020, post-data collection, an exploratory focus group was undertaken with key stakeholders within TUP (see Supplemental appendix 3).

Data analysis was done via ‘thematic analysis’ (Braun and Clarke, 2006) where data was classified into categories, reduced and arranged into manageable forms and patterns developed and substantiated. This was done by SW and AB initially independently followed by a process where thematic consensus was sought. These patterns were based on a broad ‘abductive’ approach (Dubois and Gadde, 2002) that sought to accommodate both pre-defined theoretical resources from the ‘settings’ and ‘resilience’ literature as well as grounded perspectives from informants. The work gained approval from the University of Glasgow’s College of Social Science Ethics committee.

## Findings

Following data collection and analysis derived from an amalgam of our initial research questions, theoretical foundations and grounded insights, two main themes were constructed: ‘foundational assumptions around resilience’; ‘specific TUP features associated with resilience’ and therein, a number of associated sub-themes.

### Foundational assumptions around resilience

For stakeholders, resilience was primarily conceptualised as being both a flexible and dynamic *on-going process*. In articulating such a framing, a range of analogies were offered; for example, that human beings are like “trees” or “elastic bands”, which need to bend rather than break when exposed to external stress:

“*I visualised resilience as being a kind of bendy tree in a big storm….so when the storm comes, your being battered about but actually afterwards you’re still standing*” (External associated professional); “*I suppose to me it kind of means that they have the ability to bounce back in adversity*” (External stakeholder, Parent of Trainee).

Furthermore, this flexibility was seen in the context of the particular challenges that young people with intellectual disabilities potentially face - a form of “*challenge*” (TUP Mentor) and wider “*adversity*” (TUP Senior Staff) whereby an individual potentially perseveres and prevails via “*standing up*” (TUP Senior Staff) and “*reacting to*” (TUP Senior Staff) these challenges. In contrast to seeing this as inescapably problematic, a Male Trainee openly articulated the perceived *value* of such challenges, “*before I came here, I wasn’t really confident in myself….my parent’s tried to help me with my confidence…..but I think it was coming here…it was like actually more challenging in a way*”.

This ground was strongly associated with a degree of socially embedded *inevitability* – as a “*part of life*” (TUP Senior Staff) in *“the big bad world*” (TUP Mentor). A senior TUP stakeholder summarised this ground as, *“it’s…about trauma….and just challenges that you face every day…how you deal with them”*. This foundational conceptualisation was complemented with more grounded responses associated with resilience, variously: “*a coping mechanism*” (TUP Mentor); “*having confidence*” (TUP Mentor); “*keeping self-esteem*” (TUP Senior Staff); and most practically “*not panicking*” (TUP Mentor).

Coupled with such conceptualisations were the views that resilience is closely associated with multiple expressions of ‘*independence*’. This was repeatedly expressed in various forms: “*independence and resilience go hand in hand*” (TUP Mentor); “*that you can’t have resilience….if you don’t believe that you can manage independent living*” (TUP Mentor) and “*any kind of definition of resilience connects to at least having some independence*” (TUP Senior Staff). Significantly, as well as being linked to posing abstract concepts such as “*self-determination*” (External associated professional), “*having self-belief*” (Parent) and “*managing risks*” (TUP Senior Staff), the functional context of the expressions tended to be relatively pragmatic, related to: travel [“*getting the bus…to go somewhere …..independent of that support structure around*” (TUP Mentor)]; financial management [“*taking care of their budget*” (TUP Mentor)]; domestic work [“*living independently…..cooking a pot of soup…. washing the toilet*” (TUP Senior Staff)] and socialising [“*go out clubbing….go and have a drink….go and do what everybody else does*” (TUP Mentor)]. This ground was ultimately linked to a deeper maturation process - transitioning from adolescence to adulthood in, “*be adults….take risks*” (Parent). Stakeholders felt that such a transition was critical if one is to face the inevitable ‘challenges’ of life.

Finally, resilience was occasionally seen as an *internalised* concept, focussing on the self, expressed as, “*I think that’s a self, an inner thing that builds up resilience”* (TUP Mentor) but moreso, a consequence of ‘nurture’, for example, “*it’s not something you are born with… it’s something you grow in to*” (TUP Mentor) and “*like parenting it depends on the nurture…the adults round about you*” (Parent).

### Specific TUP features associated with resilience

A number of crucial resources within TUP relating to the fostering of resilience were identified. For trainees, *personal support* was seen as most immediately significant in helping them through difficult times and was most often associated with overcoming relatively practical, even mundane tests within the community café setting, such as dealing with difficult customers, for example a Female Trainee felt

*“It was my first time on drinks and cakes, and I was like, I’m not coming back here, but [my mentor] kept putting me on it and she was like, ‘I know you don’t like it but it’s training’, and then I just like it, and it’s alright now … I’m getting used to it”*.

This ground was often associated with the active development of “*confidence*” (Male Trainee), which trainees felt helped them with tasks they had previously found challenging (like for example, gaining vocational qualifications), and more daunting tasks that they previously thought impossible, (such as gaining employment or taking part in community events alongside professionals and elected politicians): “*the fact that they stood, stood in and still believed in me when I was going through a difficult time helped*” (Male Trainee) *and* “*she’s [her mentor] been helping me to get my nerves out the way and talk more*” (Female Trainee).

As well as support derived from TUP staff and mentors, peer support and natural friendship within the group was highlighted by trainees as being important – often simply seen as the opportunity to have “*a bit of banter*” (Male Trainee). It was clear that for some trainees joining TUP, this was the first opportunity they had to develop such supportive relationships in a professional environment and to gain support from it, for example:

“*it’s just loads of fun….meeting pals*” (Female Trainee);

“*TUP is a bit different, when I worked somewhere else, obviously you can’t have a laugh like you can here”* (Male Trainee);

“*the same with friends though, a lot of these youngsters come here and have no friends. So, and friendships build for them here as well. And that’s kind of, you know they have a wee social side to it*” (TUP Mentor).

Whilst trainees perhaps naturally saw personal support in relatively unilateral and one-dimensional terms, stakeholders conceptualisation of it was more multifaceted. As well as the solely affirmative supportive base suggested above, they also saw a more robust dimension to this, centring on what personal support was *not* – “*molly-coddling*” (TUP Senior Staff), “*smothering*” (TUP Mentor) or being “*wrapped in cotton wool*” (TUP Senior Staff).

This belief was based on a concern for those providing support being potentially overprotective and that in the long term, this may hinder exposure to realistic risk and ultimately hinder the development of the trainee (in both employability and wider wellbeing terms). From their perspective, personal support should provide a basis for trainees to extend beyond self-perceived boundaries. For example, it was suggested that: “*it is really important for us…. we in a controlled manner push them….their boundaries….the fact that they probably have been told their whole life that they are not going to achieve anything…so we then push those boundaries*” (TUP Mentor). The consequence of this forceful approach was expressed by a Female trainee as, “*I think I just felt comfortable when I was in there….and I was like….let’s just try it*”.

Beyond this support, the significance of TUP as a structural and cultural “*safe space*” (TUP Mentor) was prominent, particularly from a trainee perspective. Trainees made it clear that within the setting, they genuinely “*felt safe*” (Female Trainee) and that compared to previous workplaces they could, “*breathe more*” (Male Trainee). This feeling of security starts to allude to a deeper collective notion based a principle of solidarity, an Internal stakeholder (TUP Senior Staff) expressing it as, *“rather than it’s an argument to you personally, it’s about an argument to the situation that they find themselves in…and you just happen to be that person there taking the brunt of that argument”.* Furthermore, this suggests the idea of TUP is a ‘whole system’ nurturing community with ‘the organisation’ as the core unit rather than the individual, described as involving a “*total sense of belonging*” (TUP Senior Staff).

This ‘safe’ orientation existed alongside a complementary theme advanced by certain informants that emphasised the importance of helping trainees understand and foster their potential to extend them *beyond* their ‘safe’ boundaries. This was articulated as a notion for example of “*breaking through their comfort zones*” (TUP Mentor) and was often seen as an inevitable and embedded consequence of the “*naturalistic*” (External associated professional) and “*front facing*” (TUP Senior Staff) ‘real world’ setting that is the TUP community café and where expectations are that service would be the same as any other commercial café. An associated feature of this context was the notion of the trainees being set high standards and the expectancy of “*professionalism*” (TUP Senior Staff) around the work that they did – captured by an Internal stakeholder, when they described their TUP Mentor as being “*good strict*”.

The crucial element of such a process was the *experiential* nature of tasks within the café where trainees faced a degree of stress, flux and unpredictability that allowed them to experience and manage real life uncertainty; for example, “*the resilience that comes from your angry customers…. your boredom on the downtime and the stress of the big busy times…you need to be resilient within yourself almost every single day*” (TUP Mentor). In more specific terms, this controlled insecurity was furthered in the organisation’s rota system, “*they (trainees) work with different people every day…if we allocated them a mentor…they would not take instructions from any other mentor….and if that mentor was on holiday or it was not one of their working days…it would create havoc in the café because they wouldn’t take instruction from anybody…so again…in resilience and their own ability to work with other people….is that they are allocated a different mentor on a day-to-day basis*” (TUP Senior Staff).

This latter flux was also related to the existence of a relatively high degree of organisational flexibility. Some trainees pointed to deficiencies in confidence amongst trainees that resulted in them not being able to work in certain parts of the café doing particular tasks when first joining TUP. However, adopting a bespoke ‘horses for courses’ approach, they were allowed to choose where they wanted to work, which gave them the opportunity to undertake roles which met their immediate capabilities/needs; for example, “*we find out what you enjoy….what you are good at….that’s where you can choose to excel…give them the opportunity to try all the areas in the café*” (TUP Mentor).

Accordingly, for some stakeholders, by being exposed to these new and novel situations on the job through “*tailored exposure*” (TUP Senior Staff), it was felt that trainees learn how to negotiate risk and uncertainty in a controlled yet authentic environment, for example:

“*there is no reason why they shouldn’t be put out of their comfort zone if they’re, they’re human, every human should be given the tools to, to be able to cope with going out of your comfort zone, you can’t just say ‘oh well they’ve got a learning disability, so let’s not*” (External associated professional);

“*the mentors are there for support and advice to help through the workbooks, but they don’t do all the work for the young person because they would not then have the skills or developed the skills or developed the experiences that we want for them to be able to go on and then put these experiences into paid work environments*” (Parent).

The consequence of this realism and flexibility was that mistakes were seen as an integral inevitability of the organisation’s culture and even welcomed, for example, “*they’re allowed to kinda flourish in their own right…which means making mistakes*” (TUP Mentor).

In a related yet wider sense, stakeholders also noted the way in which TUP exposes to and thus integrates its trainees into a wider social community. Most directly, this was attributed to the fact being a ‘naturalistic’ day-to-day functioning café that also hosts a variety of additional commercial and social events, opportunities for direct interaction between young people with intellectual disabilities and the general public are possible. Thus, it was implicitly possible to show the public that these young people can successfully work in a stressful ‘real world’ context. Moreover, it offers the possibility of raising awareness of the wider contribution that those with ASN can make to society:

“*they’re going in there because it’s a space where they can have a lovely coffee…and as a result, they then get to learn that people with learning disabilities are perfectly capable of doing anything, and they’re human beings and therefore they can connect with them and vice versa. And so, its integrating into the community, it’s not segregating*” (External associated professional)

“*we are talking about a couple hundred people at a wedding, and they get on amazingly well. It’s daunting at first but then they fair enjoy it, and they want to do another one. So, to me that is kind of bringing in acceptance and their social circle*” (TUP Mentor).

In conceptualising these nurturing processes as dynamic and on-going *processes*, all of this work was crucially seen to be located in a long term progressive approach with a variety of specific features: trainees being given *regular* and *tailored* 1-1 support [“*we tailor the experience for the individual….everybody I think is getting opportunity…. (well) not everybody is getting the same opportunity….I think they’re getting the best opportunity they can for their own individual need*” (TUP Senior Staff)]; having *sufficient* time to achieve set aims [“*I think we do give people time…I think we give them time to adapt to the setting*” (TUP Mentor); and the creation of a context where they could *gradually* progress at their own speed [“*our approach is not set in stone….it’s purely a framework….we work with each individual young person on a one-to-one basis at the speed that they learn…so, as long as there is progression being seen throughout the time…then we will actually allow that young person to take as long as they needed to take*” (TUP Senior Staff)].

## Implications of the findings

In this section, we look to do two related things: first, to reflect in more theoretical depth on these at present predominantly empirically framed domains (the core conceptual nature of resilience and ‘settings’-based mechanism for its promotion); and second, to explore their potential inter-relatedness.

In relation to core conceptualisations, four features were significant within TUP: the perceived *continuous* and *dynamic* nature of resilience; the *inevitability* (‘part of life’) of pressures and threats for young people with intellectual disabilities that are embedded in both society in general and within TUP as a workplace; the preference of focussing on what their trainees *can* do rather than what they *cannot*; and the notion of ‘resilience’ being a resource that contributes to further goals associated with maturation.

The first is very much in keeping with a realignment identified by Ungar et al (2007; 287-288) from seeing the attainment of resilience as a relatively narrow, static and discrete task to a ‘second ‘wave’, that highlights ecological *processes* and the “temporal and relational aspects of positive development”. In this sense, in the context of the never-ending demands of delivering a working café, the day-to-day, ongoing desire for TUP to promote relationship building within the trainee group and between trainees, staff and the public was particularly significant.

The second is a direct challenge to the hedonic and affirmative approaches of the ‘positive psychology’ and ‘wellbeing’ movements (Kahneman, 1999) that tend to seek to wish away the possibility of the existence of adverse forces in various parts of people’s lives (Wong and Roy, 2018) in favour of one-sided, largely *individualistic* (non-ecological) and arguably ‘over-protective’ approaches (Ruggeri et al, 2020). Again, the way that TUP actively exposing their trainees to situations of controlled risk conforms to this ethic.

The third is located within an ‘enablement’ ethic, comprised of various features like, “active listening, good communication, collaboration, egalitarian relationship, consideration of the person as a whole, individualized teaching, valorisation of the person’s strengths” (Hudon et al, 2011; 145), and of particular relevance to our ‘settings’ approach, undertaken in a “favourable environment, positive atmosphere: climate of mutual trust and respect, adequate time” (Hudon et al, 2011; 145). As well as this ‘challenging’ ethic, in a complementary fashion, TUP reflects this ground, having a strongly affirmative foundation within its cross-organisational values.

The fourth is suggestive of a congruous dynamic within deliberations around the concepts of ‘health’ – and the problem of ‘healthism’ (Crawford, 1980), where health becomes an end in itself (rather than foundational means to a deeper end (Seedhouse, 2001). In TUP, resilience as a ‘thing’ was rarely cited, its existence implicitly embedded in *embodied* organisational cultures and practices (Küpers, 2013) and whose existence was intrinsically linked to successfully running a working café, as well as to deeper social goals like maturation and social inclusion (as has been suggested by Feldman, 2020).

The ‘wave’ that Ungar et al (2007) refer to above also accommodates the orientation we have alluded to around notions of ‘agency’ and ‘determinism’ in fostering resilience (Connor and Zhang, 2006) and TUP suggested a highly nuanced orientation towards this dynamic. Whilst the particular importance of *environments* for those with intellectual disabilities (Vlot-van Anrooij et al, 2020c) and the resulting significance of the notion of ‘settings’ (Vlot-van Anrooij et al, 2020b) and ‘supportive environments’ (Kaplan and Kaplan, 2003) was a prominent and possibly the predominant ethic for their work, expectations of individual ‘agency’ in their trainees was never lost and was strongly evident, reflected for example in the notion of them possessing high standards and the expectancy of “professionalism” and “*good* strict”.

As suggested above, this was linked to notions of ‘high expectations’ related to the potential of the young person as well as more pragmatically as a simple pre-requisite for the successful functioning of a ‘professional’ commercial café. As such, this circumstance was highly congruent with Ungar’s ‘negotiation’ between individual needs and contextual resources and as such ‘unique pathways’ (Ungar et al, 2007; 301). In this context, we were able to recognise TUP activity that conformed to the various themes that define these complex systemic interactions, variously: via the fundamental employment-based nature of their enterprise, they provide ‘*access to material resources*’; the core inclusive and participatory ‘whole community’ culture of the organisation promotes ‘*relationships with others*’; the collective sense of the café as a holistic organisation based on ‘strengths’ fosters ‘*individual identity*’ and ‘*cultural coherence*’; and most profoundly, via their participative and political ethics associated with the natural interaction with the ‘real’ customers in a ‘front facing’ café and beyond in relation to their connectedness to wider political, employment and education partners, a sense of ‘*power and control*’ and ultimately ‘*social justice*’ in challenging longstanding prejudices and historic forms of exclusion (Thorn et al, 2009). These features are all particularly significant given the rural setting of the organisation and as such, the recognised potential for these circumstances to limit opportunity for young people with intellectual disabilities (Odiyoor and Jaydeokar, 2019).

These ‘tensions’ fed through to more grounded experiences in the actual day-to-day running of this ‘naturalistic’ café. Within a long-term, progressive and bespoke-oriented approach, we observed the striking of intricate balances between what could be consider opposing stances at various levels; continually negotiating boundaries between freedom, choice, agency, expectation and responsibility. On the one hand, TUP clearly sought to provide a safe, supportive and at times even protective environment whilst also accepting inevitable uncertainty and challenging trainees to move *beyond* their ‘safe’ boundaries and break through ‘comfort zones’. These features existed in what can be seen as a *dynamic interplay* between active tailored support when needed and independence couched in the notion of “*stand back mentoring*” (TUP Mentor). This involved assessment and negotiation between mentors and trainees on an ongoing, often minute-to-minute basis. These themes are summarised in Table 3 below.

**Table 3.** Dynamic tensions in the delivery of support associated with resilience.

	‘Protective’ features		‘Challenging’ features
*Level of assistance*	Active, interventionist support (when needed)	⇔	Fostering independence and taking responsibility (and the notion of ‘stand back mentoring’);
*Orientation of support*	Tailored individualised approach	⇔	Recognising the collective needs of the whole organisation as a working café
*Degree of consistency*	Providing consistency and security through regularity and routines	⇔	Recognising the importance of uncertainty and unpredictability as a pre-requisite of resilience
*Nature of spaces*	Creating safe and secure spaces	⇔	Allowing and enabling spaces to be unpredictable where trainees go outside their comfort zone

Whilst those with a more rationalist orientation may see these positions as conceptually contradictory and practically confusing (Rothbart and Park, 2004), there is a growing sense that ‘ambiguity’ is both inevitable (Berndt and Sachs-Hombach, 2015) and potentially creative and constructive in practice communities (Kaethler, 2019). In broader terms, this ground can be seen again in relation to our earlier contention that wellbeing and resilience-oriented interventions tend to feel the need to be wholly affirmative in their approach, under-representing the existence of potentially challenging influences. Rather, the TUP ethic suggested the need for the expression of *some* antagonistic ingredients and ‘risk’ as necessary and possibly essential pre-determinants of fostering resilience. This can be best located in relation to three deeper inter-related notions: the significance of a profound and complex ‘eudaimonic’ orientation that frames wellbeing as more than simple hedonic ‘happiness’ (Huta and Waterman, 2014); the existence of ‘salutogenesis’, where ‘health’ is expressed through coping with flux and stress, specifically, “a life experience of bringing resources to bear on coping with stressors that shapes the sense of coherence” (Mittelmark and Bauer, 2016; 8); and as such, in a wider sense, the need move beyond ‘risk averse societies’ (Gill, 2007) and embrace ‘risk oriented’ experiences and interventions (Cooke, Wong and Press, 2021).

## Conclusion

In practical terms, our work has shown that TUP provides a conducive ‘ecological’ organisational context whose features have the potential to foster resilience. We have also begun to understand how in ‘on-the -ground’ circumstances, the TUP model successfully negotiates a constructive dynamic between ‘support’ and ‘exposure’. The conceptual bases we have drawn upon in founding our practical approach – enabling and embodied ‘ecological’ approaches to resilience with a ‘settings’ approach - have attracted critique for their tendency to couched in theoretical and aspirational rather than practical terms (Hall and Theron, 2016; Dooris, Wills and Newton, 2014). Naaldenberg et al. (2013; 4541) has also suggested a series of ‘challenges’ in implementing any ‘health promotion’ intervention with those with intellectual disabilities, centring predominantly on a lack of specialist expertise in programme delivery, poor resourcing and capacity, low levels of genuine participation and disinterested organisations. Our grounded insights begin to illuminate these gaps and the embedded and embodied nature of TUPs work goes some way towards addressing these issues; particularly, integrating activity in a person’s natural setting and cultivating high levels of participation. In being based on social, political, organisational, psychological and educational principles, it is also suggestive of the potential for the interdisciplinary approaches in this domain.

Nevertheless, our work is clearly open to critique in both methodological and political terms. Practically, it is a small scale ‘pilot’ of one particular organisation. The formal data collected came largely from secondary accounts rather than observed practice itself and from a relatively limited sample. By focussing only on TUP as a distinct organisation, we were also unable to assess the impact of influences on resilience amongst trainees beyond this defined context. Concerns for core validity, wider generalisability and translation to other settings must therefore be recognised.

However, decisions we made and associated features of the work go some way towards mitigating against these concerns. Our internal sample was relatively wide, accommodating a variety of informants (trainees, mentors and organisation management). We also sought to allude to their wider social experiences by accessing views from parents and associated professionals. Our project Research Assistant also volunteered as a mentor in the café prior to the interviews, thus gaining deep insight into grounded everyday activities. Finally, we purposefully based our data collection and subsequent analysis on a series of established theoretical resources that allows us to have some confidence that a degree of *theoretical* generalisability was attained (Carminati, 2018).

In terms of wider political critique, the coupling of ‘resilience’ particularly to ‘employability’ in a ‘neoliberal’ context is considered by some as potentially problematic (Tierney, 2015). Such perspectives conceptualise this as a predominantly ‘supply side’ approach to employability, where individuals are obliged to ‘fit’ into existing workplace demands (Peck and Theodore, 2000) and where employment ‘resilience’ may be the only option in harsh ‘free market’ environment (Dowse, 2009). As such, in terms of resilience, these circumstances might in principle possibly expose young people with intellectual disabilities to excessive ‘challenges’ with insufficient protection and support.

However, building on themes identified specifically in this project, we argue that mitigation is possible. First, employability practice need not *necessarily* be problematic. For example, Kin Kwan (2019; 2) advances the possibility of “socially responsible human resource practices” for those with disabilities in ‘mainstream’ employment, suggesting a series of associated practical features, including for example, educational exchanges, tailored approaches and various forms of ‘accommodation’ (Fasciglione, 2015). In of itself, TUP conforms to these principles and actively engages with their trainees end-destination employers (Whitelaw et al, 2021). Second, many point to the potential that employment has in conferring social status and fostering resilience; both generally (Hartung and Cadaret, 2017) and in relation to those with intellectual disabilities (Kirsha et al, 2009). In Ungar’s (2008; 231) terms, employment can be seen as one prominent feature in the “cultural practices, values and beliefs” that shape a market-based western society and economy and as such offer a form of ‘cultural adherence’ for young people with intellectual disabilities. Moreover, some see employment as an inherently political act, a means of challenging disability stereotypes and fostering social inclusion via workplace experiences (Barnes and Mercer, 2005). Again, TUP has engaged at local (businesses and community groups) and national (Scottish and UK Governments) levels to challenge systemic employability barriers and social attitudes (Whitelaw et al, 2021).

Beyond this pilot, we suggest the following further pieces of work ‘next possible steps’: a more detailed examination of *how* specifically organisations might nurture and enact such a culture that fosters resilience in terms of organisational governance; a more detailed examination of the *specifics* of a form of practice that successfully negotiates the constructive tension between ‘exposure’ and ‘support’ in fostering resilience; an exploration of the extent to which TUP specific resources and approaches have the potential to be translated into other types of organisational settings – for example, schools or workplaces.

## Supplemental Material

Supplemental Material - Fostering resilience in young people with intellectual disabilities using a ‘settings’ approach Supplemental Material for Fostering resilience in young people with intellectual disabilities using a ‘settings’ approach by Sandy Whitelaw, Anthony Bell, Ailsa Mackay and Heather Hall in Journal of Intellectual Disabilities
