# Supplementary material for: Fostering resilience in young people with intellectual disabilities using a ‘settings’ approach
Source: J Intellect Disabil. 2023 Mar 28;28(2):549–66. doi: 10.1177/17446295231168186 (PMC11059839; doi:10.1177/17446295231168186)
Supplement: Supplemental Material - Fostering resilience in young people with intellectual disabilities using a ‘settings’ approach [file sj-pdf-1-jld-10.1177_17446295231168186.pdf]

Appendix 1: Narrative Interviews -Topic Guide and Prompts; Created from the theoretical work by Mabhoyi and Seroto (2019), Atkinson (1998), and Theron and Theron (2014).

	**Part 1**
**Stage/Type of Question**	**Narration Phase/Open ended**
**Question**	• So, in starting the interview, can you tell me about how your life was like before working here?<br><br>• How did you end up at TUP?
**Potential Prompts**	• Prompts could include:<br>○ *Could you find any work*?<br>○ *What was it like meeting new people at TUP?*

	**Part 2**
**Stage/Type of Question**	**Narration Phase/Contrast Question**
**Question**	• Thinking about how you have changed since then, how different is your life now than it was before you worked here?
**Potential Prompts**	• [Coffee machine example] – what about yourself, what skills have you gained or while working at TUP?<br><br>• Do you feel you have lost any skills while working at TUP?<br><br>• Can you give me some examples from your own experience?

	**Part 3**
**Stage/Type of Question**	**Conversation Phase**
**Question**	• Can you tell me how The Usual Place (TUP) has helped you to do well in life at times when you felt that life was difficult?<br><br>• Has there been any instances when TUP has not helped you when things got difficult?
**Potential Prompts**	• If possible, try and elaborate on a specific themes/points they raised **prior to this question**, and touch on it here.<br><br>• Where possible, encourage the participant to also talk about the COVID-19 lockdown (**within the frame of their narrative**), and how TUP helped them do well during such an event.<br><br>• Feel free here to **ask further question**, which are directly related to themes the participant raised throughout their narrative (try to use their phrasing).<br><br>• [*Finish with a positive theme (ideally one raised by the participant!*]

Appendix Two: Semi-structured Stakeholder Interview Guide; Created from the theoretical work by: Ungar *et al* (2007) Unique pathways to resilience across cultures. Adolescence, 42 (166); 287-310.

	**Question 1**
**Question**	• From your perspective, what does it mean to be 'resilient'?
**Potential Prompts**	• Prompts could include:<br>○ Offering silence to encourage participant reflection here.<br>○ Offer your own interpretation of resilience i.e., someone who continues to thrive in life, despite exposure to difficult life circumstances?

[Introduction and brief, following a discussion of consent and right to withdraw etc.]

	**Question 2**
**Question**	• In terms of relationships developed here at TUP, how important do you feel these are in fostering resilience among the trainees?
**Potential Prompts**	• Prompts could include:<br>○ Relationships can sometimes provide us all with emotional support and a sense of belonging – just think about how this may apply within a place like TUP (i.e., trainees and mentors).

	**Question 3**
**Question**	• What features of TUP do you feel help the trainees develop a sense of identity?
**Potential Prompts**	• Prompts could include:<br>○ Features could be more **individualistic** (such as job role or sense of self-belief).<br>○ **Collective** – (organisational ethos/values – belonging - being part of the wider community).

	**Question 4**
**Question**	• The ability to make informed decisions on your own is an important aspect of independent living. What role does TUP play (if any), in helping make its trainees more independent?
**Potential Prompts**	• Prompts could include:<br>○ Providing an example – working in the café involves making individual choices most of the time (i.e., even just how you approach customers).

Question 5 (i)	
**Question**	• What challenges do you think young people with ASN face in a 'culture' like ours, where a lot of the time people are expected to 'pull themselves up with their bootstraps'?
**Potential Prompts**	• Prompts could include:<br>○ Stigma, and the assumption that the young person will always require care?<br>○ Adopting a pathological [**problem based**], rather than salutogenic [**asset based**] approach?

Question 5 (ii)	
**Question**	• How do you think TUP challenges (or even perpetuates) such thinking?
**Potential Prompts**	• Prompts could include:<br>○ The emphasis on equality and everyone's value in the ethos of the organisation; "*Everyone is of equal worth. Everyone can contribute to their community. Everyone should be treated with dignity and respect*".<br>○ Helping people thrive, rather than expecting them to do it themselves.

Question 6 (i)	
**Question**	• How does TUP try and integrate trainees within the wider Dumfries community?

Question 6 (ii)	
**Question**	• Building from our last question, do you think having some sense of responsibility to the greater good or community helps people during challenging moments in their lives?
**Potential Prompts**	• You said that TUP gives young people a sense of belonging in their wider community context, what do you mean by that (*elaborating follow up*).

Question 7	
**Question**	• Its nearly the end of the interview, would you like to discuss anything else?

Appendix 3: Initial provisional themes: Focus Group Briefing Paper

I. Some foundational assumptions around resilience

- Basic articulation of resilience: 'independence' + as form of maturation and becoming an adult; 'flexibility'; 'cope'/'coping'; 'confidence'; 'self-esteem'; 'not panicking' - "bendy tree in a big storm….when the storm comes….you're being battered about but actually afterwards you're still standing…so if you are brittle or not resilient….then you're going to get blown over"; "like kinda *elastic band*";

- Both an **internalised** concept [an "inner strength"] and as the consequence of nurture ["*not* something you are born with…something you grow in to"; "like parenting it depends on the nurture…the adults round about you"];

- Various forms of possible ***adversity*** and possible ***trauma*** recognised and accepted up-front as a part of the domain and 'built-in' to responses;

- Seen as an ***inevitable challenge*** that is 'part of life' and a 'big bad world'.

II. Specific TUP setting features associated with resilience

- Broad notion of needing to **'stand up'** and '**react**' to **challenges -** "it's…about trauma….and just challenges that you actually face every day…how you dealt with them";

- What it is ***not***: 'molly-coddling'; 'smothering'; "wrap in cotton wool";

- Understanding and fostering *potential*, extending beyond boundaries ***and*** *limits*- "they actually understand what their parameters are…and their *boundaries*"; "helping to push them to know where their boundaries are…. getting them out of their comfort zone";

- '**Employability**' as central focal point and testbed for testing and promoting resilience;

- The centrality of **doing** and **experiential** and **situational** experiences;

- New and novel situations/dynamic situations/allowing **uncertainty/flux/unpredictability** are all fostered with different stressful situations in different contexts and sense of 'tailored exposure' to suits needs = "not panicking" and "managing uncertainties" and "allowing the bad day to happen" as basis of resilience;

- Linked to realistic **'front-facing'** café/naturalistic/social visibility notion - "working *naturally* within the café…out in the public";

- **Enablement/empowerment/participative/communication** as basis of resilience and expressed at various levels communication, learning, actual skills and political participation;

- **High expectations**/strict ("good strict");

- **Whole system context** of 'nurturing'/helping community/being valued/and wider having social value in the context of TUP/organisational inclusion ("total sense of belonging"); 'the organisation' as the core unit rather than the individual "rather than it's an argument to you personally, it's about an argument to *the situation* that they find themselves in…and you just

happen to be that person there taking the brunt of that argument…so it's about building up that resilience";

- **Long term** progressive aspect: sufficient time/regularity/follow through/small steps/ "natural progression"; 1-1 basis + progression at their speed; take as long as they need to take.

III. Series of dynamic tensions in the organisation at various levels

- Active tailored support (when needed) - independence ('stand back mentoring');
- Tailored individualised approach – the needs of the whole organisation as a working café;
- Consistency (regularity and routines) – uncertainty/unpredictability (flux and built in change);
- Summarised as continuum of freedom-choice-agency-expectation-responsibility.

IV. Possible deeper themes to explore

- Enablement;
- Embodiment;
- Fostering and management of risk experiences;
- Salutogenesis – health as coping with flux and stress.

V. Critical questions

- General comment on the validity and robustness of the themes;
- Comment on omissions/additions?;
- Dealing with the tensions involved in the concept of resilience (e.g. internal-external) delivery (the continua);
- Neo-liberal notions of 'employment' as a final achievement of human resilience – has good practical significance but what are the possible negatives of this? (Clash between practice and theory?).
